# The quality of reports of randomized clinical trials on traditional Chinese medicine treatments: a systematic review of articles indexed in the China National Knowledge Infrastructure database from 2005 to 2012

**DOI:** 10.1186/1472-6882-14-362

**Published:** 2014-09-26

**Authors:** Jinnong Li, Zhenhua Liu, Ruiqi Chen, Dan Hu, Wenjuan Li, Xiajing Li, Xuzheng Chen, Baokang Huang, Lianming Liao

**Affiliations:** Department of Oncology, Fujian Academy of Integrative Medicine, Fujian University of Traditional Chinese Medicine, Fuzhou, 350112 PR China; Department of Medical Oncology, Fujian Provincial Clinical College Affiliated with Fujian Medical University and Fujian Provincial Hospital, Fuzhou, 350001 PR China; Department of Pharmacognosy, College of Pharmacy, Second Military Medical University of China, Shanghai, 200433 PR China

**Keywords:** Traditional chinese medicine, Consolidated standards for reporting trials, Randomized clinical trial, Jadad score

## Abstract

**Background:**

The Consolidated Standards for Reporting Trials (CONSORT) are aimed to standardize clinical trial reporting. Our objective is to compare the quality of randomized clinical trials (RCTs) of traditional Chinese medicine (TCM) published in 2005–2009 and 2011–2012 according to the current CONSORT statements and Jadad scale.

**Methods:**

Data Sources: Reports on RCTs of TCM in the China National Knowledge Infrastructure database (CNKI database) for manuscripts published from 2005 to 2009 and 2011–2012. Search terms included TCM and clinical trial. Study Selection: Manuscripts that reported RCTs of TCM were included. Data Extraction: Independent extraction of articles was done by 3 authors. Disagreement was discussed until agreement was reached. According to the CONSORT checklist, an item was scored as 1 when the item was described in the paper. Otherwise the item was scored as 0.

**Results:**

A total of 4133 trials in 2005–2009 and 2861 trials in 2011–2012 were identified respectively. There was a significant increase in proportion of reports that included details of background (24.71% vs 35.20%, *P* < 0.001), participants (49.79% vs 65.26%, *P* < 0.001), the methods of random sequence generation (13.77% vs 19.85%, *P* < 0.001), statistical methods (63.00% vs 72.77%, *P* < 0.001) and recruitment date (70.14% vs 80.36%, *P* < 0.001) in 2011–2012 compared to 2005–2009. However, the percentage of reports with trial design decreased from 4.45% to 3.25% (*P* = 0.011). Few reports described the blinding methods, and there was a decreasing tendency (4.77% vs 2.48%, *P* < 0.001). There was a similar decreasing tendency on the reporting of funding (6.53% vs 5.00%, *P* = 0.007). There were no significant differences in the other CONSORT items. In terms of Jadad Score, the proportion of reports with a score of 2 was markedly increased (15.15% vs 19.71%, *P* < 0.001).

**Conclusions:**

Although the quality of reporting RCTs of TCM was improved in 2011–2012 compared to 2005–2009, the percentages of high-quality reports are both very low in terms of Jadad score. There is a need for improving standards for reporting RCTs in China.

## Background

In order to acquire effective and credible outcomes, randomization and control are essential for clinical trials. Randomized controlled trials (RCTs) provide the most reliable evidence of health care intervention and are the basis for the establishment of many medical guidelines. However, RCTs are not always reported with sufficient details or clarity, potentially hindering interpretation of results [1, 2]. For a reader to accurately evaluate the conclusion of a published report, he (she) needs complete, clear, and transparent information on the methodology and findings of the report. Unfortunately, attempted assessments frequently fail because authors of many trial reports do not describe some critical data and only limited information is available [3–5].

The CONSORT (consolidated standards of reporting trials) statement was first published in 1996, revised in 2001 and updated in 2010 by the CONSORT Group. They provide authors and editors with a checklist for a minimum set of recommendations for reporting the trial design, analysis and results [6–8]. Many studies have showed that quality of trial reporting can be improved when authors follow the checklist of the CONSORT [9–12]. The Jadad score is considered a valid and reliable tool to assess the methodological quality of a clinical trial, and has been applied throughout the medical literature [13, 14].

Traditional Chinese medicine (TCM), including herbal medicine, are widely used in China to treat a variety of diseases and used increasingly to complement conventional medical care globally. In a nationally representative U.S. survey conducted in 2002, almost 20% of adults and 75%–100% of Asian-Americans had used herbal therapies in the past year [15]. They believe the TCM and conventional medicine provides more optimal healing than conventional medicine alone [16–18]. However, in the era of evidence-based medicine, TCM has encountered a strong challenge from clinicians due to a shortage of evidence-based efficacy. Therefore, researchers have made a great deal of effort in TCM clinical studies. In the past decade, TCM RCT is avocated and a number of RCTs of TCM have been reported [19–23]. Recently many TCM researchers evaluated the quality of RCTs with TCM according to the checklist of the CONSORT [24–29]. Their studies show that the quality of TCM RCTs is generally low. However, these studies evaluated only one or several TCM journals, or evaluated publication on a specific disease. Thus they cannot give a comprehensive view on the overall quality of TCM RCTs.

The purpose of the present study was to compare the change in quality of reporting TCM RCTs prior to and after the publication of the 2010 CONSORT statement. We include all publications of TCM RCTs during this period in the CNKI database, aiming to comprehensively evaluate the overall quality of TCM RCTs.

## Methods

### Search strategy

The China National Knowledge Infrastructure (CNKI) database is the most comprehensive full-text database of journals published in China and was used in the present study [30]. The CNKI database has several subdatabases. Among them is the academic journals’ full-text database, which was used in the present study. We chose manuscripts published in 2005–2009 and 2011–2012, which respectively represent publications before and after the 2010 CONSORT statement. We used an electronic search strategy that involved subject term ‘traditional Chinese medicine’ and ‘clinical trial’ and “Fuzzy Search” method so as to acquire more potential manuscripts. To evaluate the tendency of publication quality, we evaluated the published reports on an annual base. The titles, index terms, and abstracts of the identified manuscripts were read and rated as “potential manuscript” or “not relevant”. We retrieved all potential manuscripts and reviewed their full texts according to the following criteria:

Inclusion criteria were manuscripts reporting TCM RCTs.

**Figure 1:**
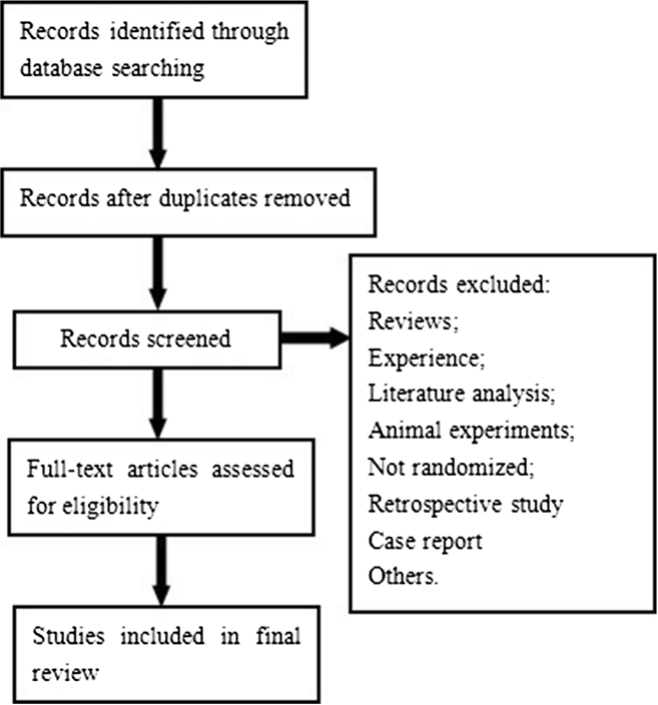
**The process of collecting materials.**

Exclusion criteria were (1) review, literature analysis, experience, case report; (2) animal experiments; (3) Non-randomized clinical trials; (4) reduplicative reporting; (5) retrospective study; (6) others. Three reviewers (J L, Z L, R C) reviewed the texts of the manuscripts to identify TCM RCTs. Disagreements regarding inclusion were resolved by discussion. Figure 1 shows the process of collecting materials and analysis.

### Scoring according to CONSORT

A checklist of 25 items from the updated 2010 CONSORT guidelines was used [31–33]. Among the 25 items, 12 have 2 subitems. The score for each item or subitem was either 0 or 1: 0 indicates no description of the corresponding item/subitem and 1 indicates there was description of the item/subitem in the report. We did not include the following subitems in our report because we found after analysis of all manuscript that (1) there were no reports that changed the methods after trial commencement (Subitem 3b); (2) there were no reports that changed trial outcomes after the trial commenced (Subitem 6b); (3) there were no reports that had interim analyses and stopping guidelines (Subitem 7b); (4) there were no reports that were stopped prematurely (Subitem 14b); (5) there were no reports that had additional analyses (Subitem 18). After these 5 subitems were excluded, the maximum score a paper could obtain 31 points. Each article was assessed for every item according to the checklist [29] by three investigators independently (J L, Z L and R C). When there were different opinions between three investigators, they discussed them until reaching a consensus. Otherwise the final decision was made by L L. The total score of each trial was calculated.

### Scoring according to Jadad

The Jadad scale is a 5-point scale for measuring the quality of randomized trials. A score of three points or more indicates high quality [13]. The Jadad scale includes how generation of random sequence is described (0 = no description; 1 = inadequate description; 2 = adequate description); how the blinding is carried out (2 = double-blinding with adequate description; 1 = double-blinding with inadequate description; 0 = wrong usage of double-blinding), and why and how often withdrawal of patients happens (When the numbers and reasons of withdrawal and exit of patients were reported, we recorded 1. Otherwise, 0 was recorded). Similarly, the work was done by three investigators (J L, Z L and R C) separately. Disagreement was discussed by three until agreement was reached. Otherwise final decision was made by L L.

### Statistics

Pearson χ^2^ test was used to test whether differences among two periods (2005–2009 and 2011–2012) were statistically significant in terms of mean total score of CONSORT. Wilcoxon rank sum test was used to test differences of Jadad scores of the different years. The levels of significance for all tests were set at 0.05. Data were analyzed using SPSS version 18.0. The total score of each report and the percentage of different score were calculated.

## Results

### Characteristics of selected RCTs

After screening the titles, abstracts and texts, we identified a total of 4133 reports in 2005–2009 and 2861 in 2011–2012 in the CNKI database that met the inclusion and exclusion criteria and were included in this analysis. The annual numbers of reports identified in each screening step are shown in Table 1.

**Table 1:** **Results of screening for randomized clinical trials from 2005 to 2012**

	2005	2006	2007	2008	2009	2011	2012
Records identified through database searching	2040	2142	2795	3595	3706	6072	5874
Duplicated papers	3	6	6	22	80	24	83
Reviews	347	387	609	773	717	1176	1255
Experience	148	146	209	242	275	785	530
Literature analysis	30	36	26	39	59	93	72
Animal experiments	22	20	17	20	18	15	12
Not randomized	512	540	648	679	772	1083	1076
Retrospective study	3	13	16	25	17	23	86
Case report	20	38	47	80	77	189	156
Others	244	257	548	704	648	1342	1085
Full-text articles assessed for eligibility	711	699	669	1011	1043	1342	1519
Studies included in final review	711	699	669	1011	1043	1342	1519

### The CONSORT results

#### CONSORT: title, abstract, background and objectives

**Figure 2:**
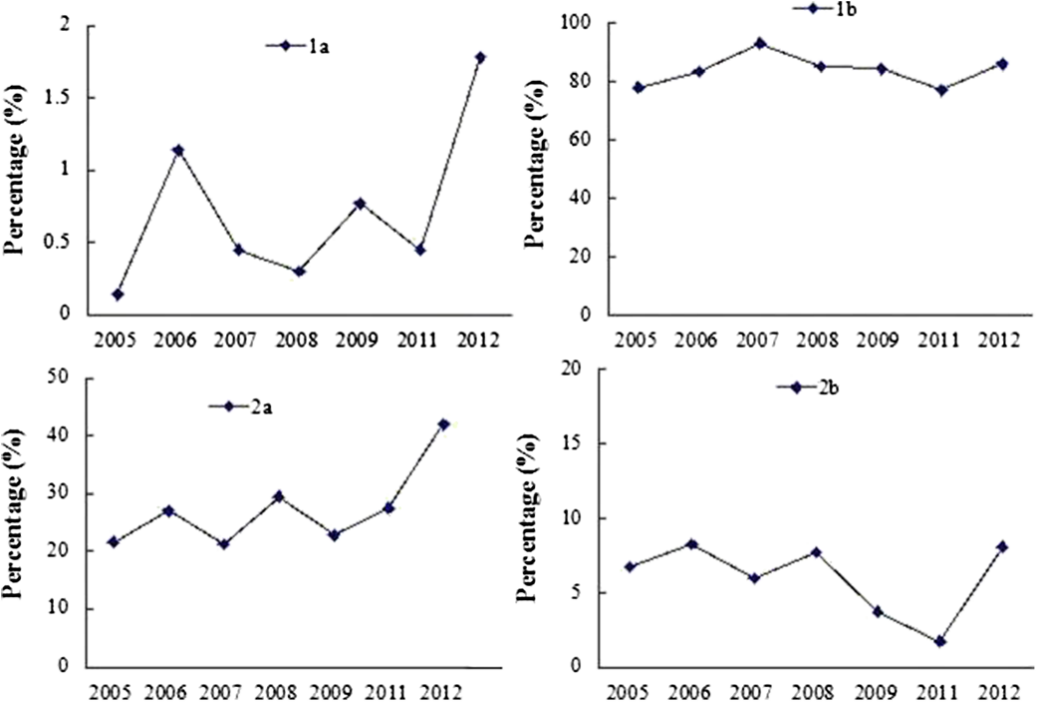
**CONSORT results of title, abstract, background and objectives in each year.**

The proportion of reports with “randomized” in the title (1a) increased significantly (0.56% vs 1.15%, *P* = 0.006). However, the percentages were very low for both periods (Table 2 and Figure 2). 84.81% of reports had abstracts (1b) that included objective, methods, results and conclusions in 2005–2009, more than that in 2011–2012 (82.03%). The proportions of reports with detailed description of backgrounds (2a) of studies were low for both periods, but were higher in 2011–2012 (24.71% in 2005–2009 vs 35.20% in 2011–2012, *P* < 0.001). The proportions of reports with objectives (2b) were also low (6.36% vs 5.14%, *P* = 0.032) (Table 2).

**Table 2:** **Comparision of randomized control trials indexed in CNKI database before and after 2010 in terms of CONSORT items**

Criterion	Item no.	No. (%) of trials in which the item was clearly reported			***P***value
		All (n = 6994)	Before 2010 (n = 4133)	After 2010 (n = 2861)	
Title and abstract	1a	56 (0.80)	23 (0.56)	33 (1.15)	0.006
	1b	5852 (83.67)	3505 (84.81)	2347 (82.03)	0.002
Background and objectives	2a	2028 (29.00)	1021 (24.71)	1007 (35.20)	<0.001
	2b	410 (5.86)	263 (6.36)	147 (5.14)	0.032
Trial design	3a	277 (3.96)	184 (4.45)	93 (3.25)	0.011
Participants	4a	3925 (56.12)	2058 (49.79)	1867 (65.26)	<0.001
	4b	4938 (70.60)	2664 (64.46)	2274 (79.48)	<0.001
Interventions	5	5699 (81.48)	3342 (80.86)	2357 (82.38)	0.107
Outcomes	6a	6176 (88.30)	3626 (87.73)	2550 (89.13)	0.074
Sample size	7a	20 (0.29)	8 (0.19)	12 (0.42)	0.082
Sequence generation	8a	1137 (16.26)	569 (13.77)	568 (19.85)	<0.001
	8b	117 (1.67)	78 (1.89)	39 (1.36)	0.093
Allocation concealment mechanism	9	48 (0.69)	30 (0.73)	18 (0.63)	0.63
Implementation	10	36 (0.51)	31 (0.75)	5 (0.17)	0.001
Blinding	11a	268 (3.83)	197 (4.77)	71 (2.48)	<0.001
	11b	8 (0.11)	5 (0.12)	3 (0.10)	1.000
Statistical methods	12a	4686 (67.00)	2604 (63.00)	2082 (72.77)	<0.001
Flow diagram	13a	20 (0.29)	11 (0.27)	9 (0.31)	0.709
	13b	246 (3.52)	141 (3.41)	105 (3.67)	0.564
Recruitment	14a	5198 (74.32)	2899 (70.14)	2299 (80.36)	<0.001
Baseline data	15	445 (6.36)	232 (5.61)	213 (7.44)	0.002
Numbers analyzed	16	127 (1.82)	80 (1.94)	47 (1.64)	0.367
Outcomes and estimation	17a	89 (1.27)	58 (1.40)	31 (1.08)	0.241
	17b	21 (0.30)	8 (0.19)	13 (0.45)	0.050
Harms	19	1830 (26.17)	1096 (26.52)	734 (25.66)	0.42
Limitations	20	572 (8.18)	356 (8.61)	216 (7.55)	0.11
Generalizability	21	187 (2.67)	115 (2.78)	72 (2.52)	0.498
Interpretation	22	6473 (92.55)	3806 (92.09)	2667 (93.22)	0.077
Registration	23	1 (0.01)	0 (0)	1 (0.03)	0.853
Protocol	24	1 (0.01)	0 (0)	1 (0.03)	0.853
Funding	25	413 (5.91)	270 (6.53)	143 (5.00)	0.007

### CONSORT: materials and methods

**Figure 3:**
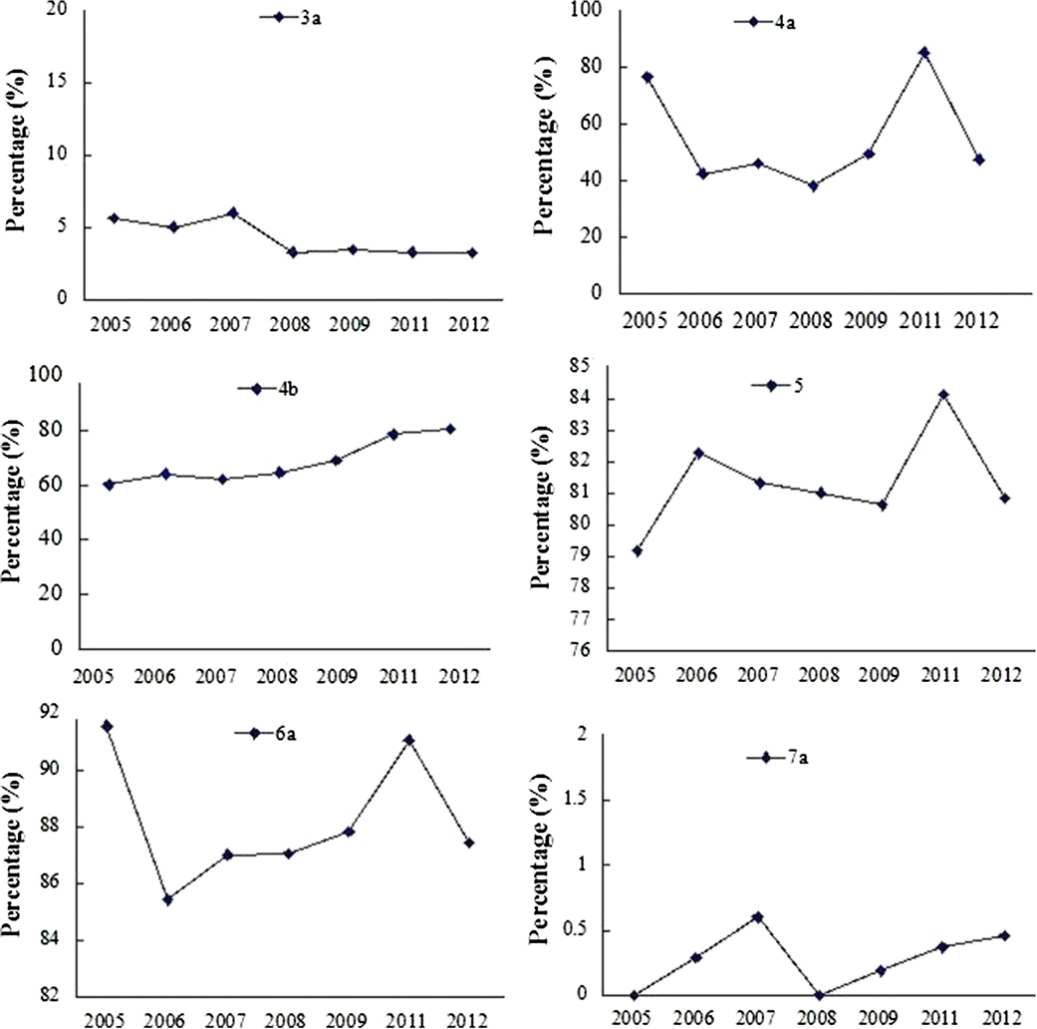
**CONSORT results of materials and methods in each year.**

Description on the following items had obvious improvement in 2011–2012 over 2005–2009: inclusion and exclusion criteria of patients (4a) (65.26% vs 49.79%, *P* < 0.001), the place of collecting materials (4b) (79.48% vs 64.46%, *P* < 0.001). However, the proportions on the description of the patient distribution (3a) decreased (*P* = 0.011). Although the proportions on the description of interventions, outcomes and the calculated sample size were improved, there was no significant difference (Table 2). As shown in Figure 3, there is a fluctuation in the proportions on the description of these items during 2005–2012.

### CONSORT: randomization

**Figure 4:**
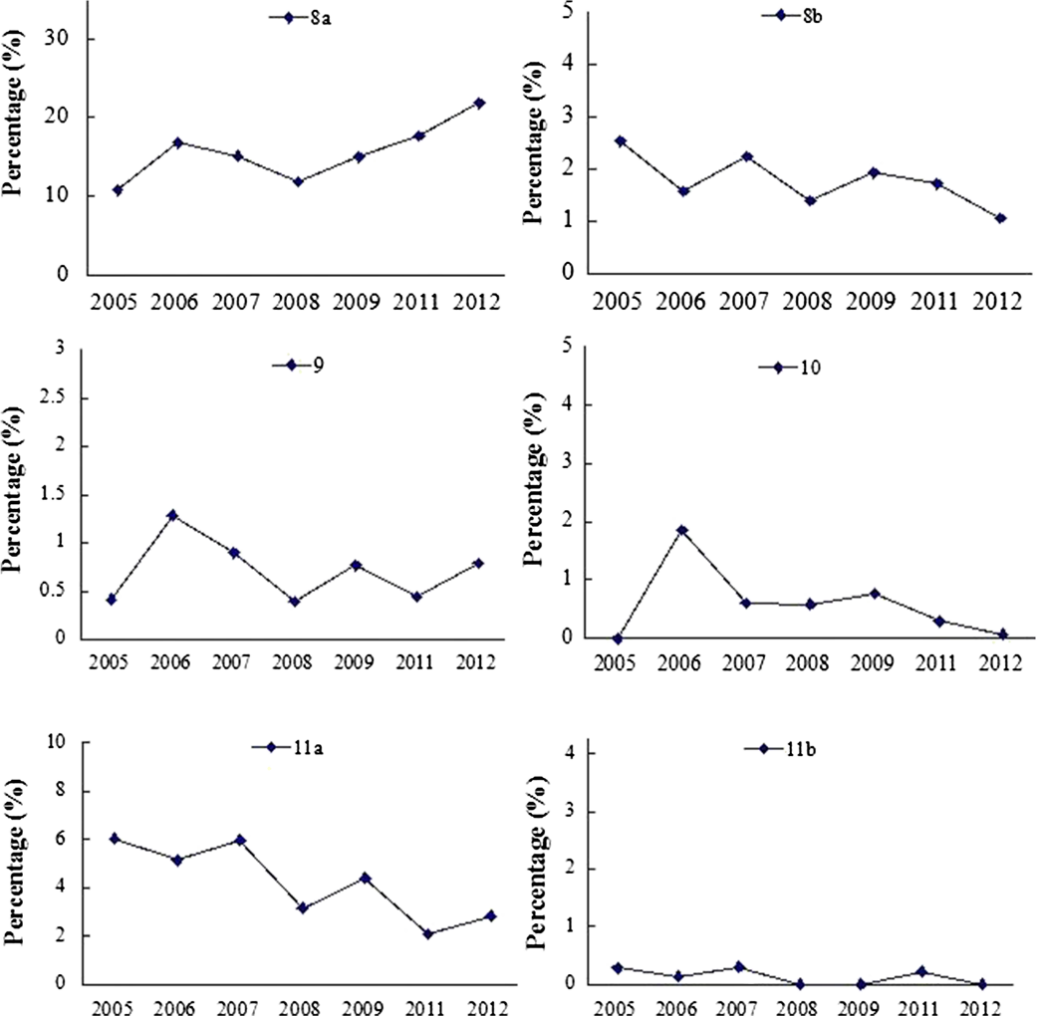
**CONSORT results of ‘randomization’ in each year.**

Description on sequence generation (8a) also increased significantly (13.77% in 2005–2009 vs 19.85% in 2011–2012, *P* < 0.001). However, the proportion of reports with blinding (11a) decreased in 2011–2012 (4.77% in 2005–2009 vs 2.48% in 2011–2012, *P* < 0.001). Similar trend was observed for description on detailed implement process (10) (0.75% vs 0.17%, *P* = 0.001). Few reports described the allocation concealment mechanism (9) (Table 2). As shown in Figure 4, there is a fluctuation in the proportions on the description of these items from 2005 to 2012.

### CONSORT: results

**Figure 5:**
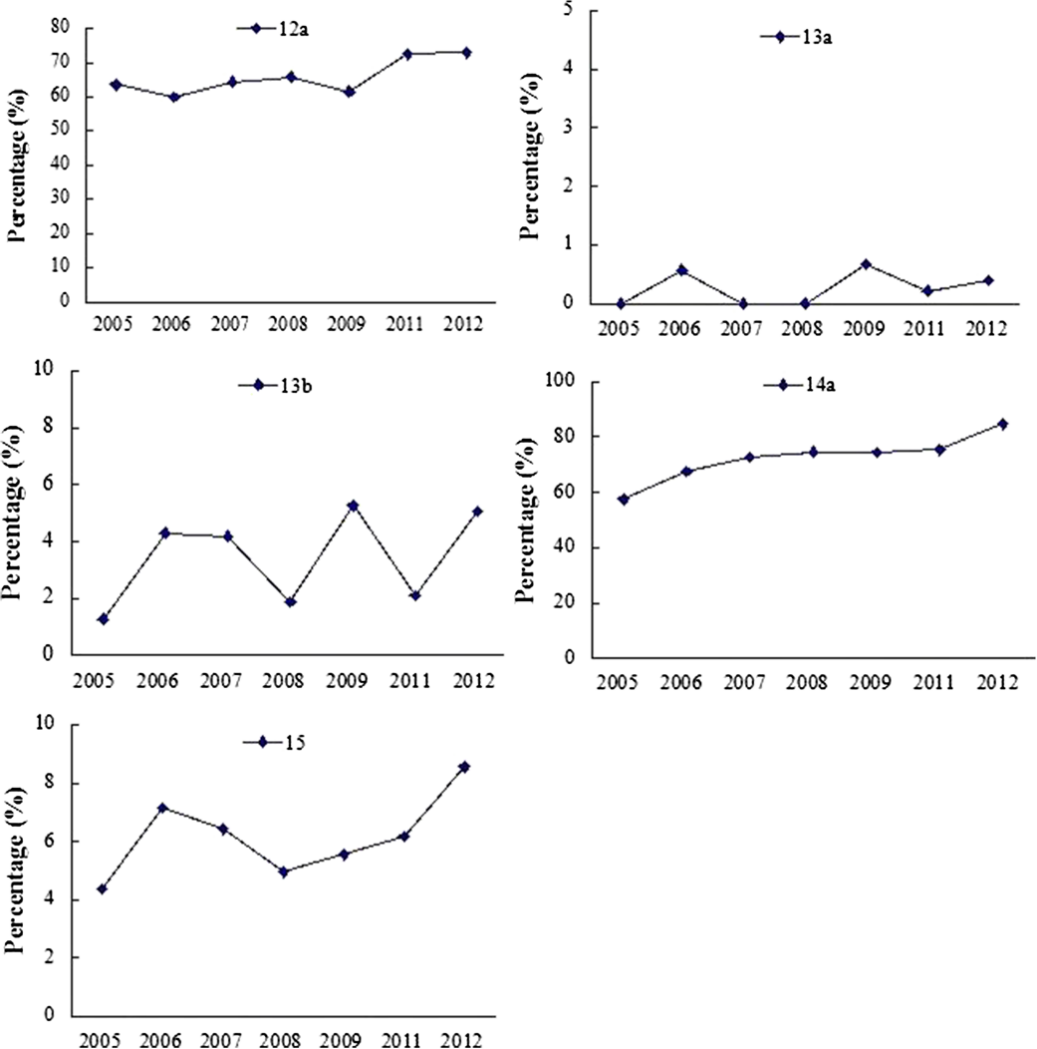
**CONSORT results of ‘results’ in each year.**

The proportion with detailed statistical methods (12a) was greater after 2010 (63.00% in 2005–2009 vs 72.77% in 2011–2012, *P* < 0.001). The proportion of reports with the dates of recruiting and follow-up (14a) was greater after 2010 (70.14% in 2005–2009 vs 80.36% in 2011–2012, *P* < 0.001). Although the proportion of papers that reported loss to follow-up (13b) and flow diagram (13a) increased after 2010, the quality remained to be improved. The proportion of reports with baseline data description (15) increased (P= 0.002), (Table 2). As shown in Figure 5, there is a fluctuation in the proportions of reports with the description of these items from 2005 to 2012 except recruiting and follow-up (14a).

### CONSORT: discussion

**Figure 6:**
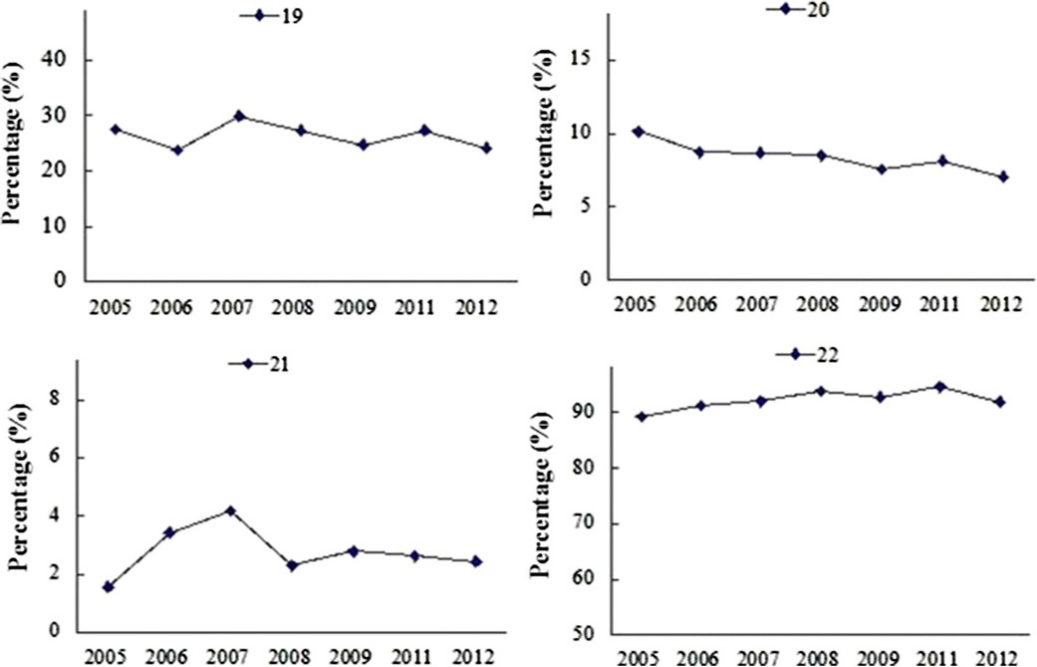
**CONSORT results of ‘discussion’ in each year.**

There was no difference in proportions of papers reporting harms (19), limitations (20), generalizability (21) and interpretation (22) before and after 2010 (Table 2). As shown in Figure 6, there is a fluctuation in the proportions on the description of these items from 2005 to 2012.

### CONSORT: other information

Only one paper reported the registration (23) or the protocol (24). The proportion of paper reporting fundings (25) decreased markedly during 2011–2012 compared to 2005–2009 (*P* = 0.007) (Table 2).

### CONSORT: total score of each report

**Figure 7:**
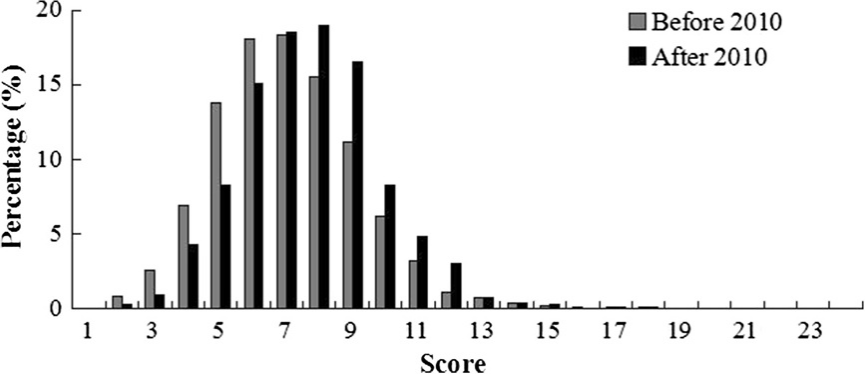
**The distribution of the mean scores before and after 2010.**

**Figure 8:**
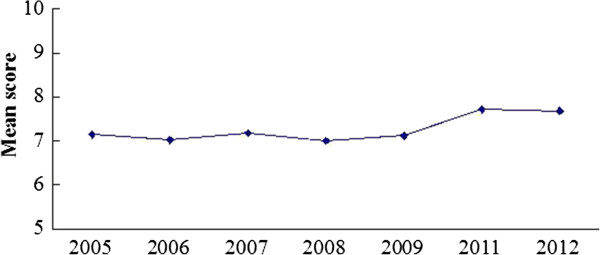
**The mean score of publication of each year.**

**Figure 9:**
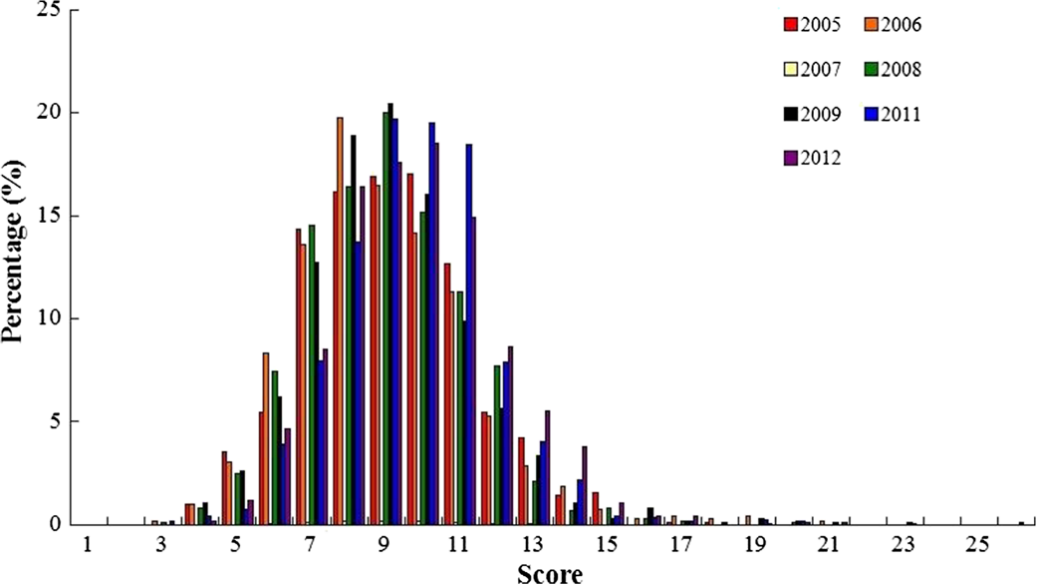
**The annual distributions of the CONSORT score of reports.**

Figure 7 shows the distribution of the mean scores of reports before and after 2010, with 24 being the highest score. The scores range from 1 to 24, with most of them within the range of 4–11. Generally the scores of reports are low for both periods and the mean score of 2011–2012 is slightly higher (7.09 in 2005–2009 vs 7.70 in 2011–2012) (Figure 8). Figure 9 shows that the annual distributions of the reports with a specific score in each year are similar.

### Jadad score

**Figure 10:**
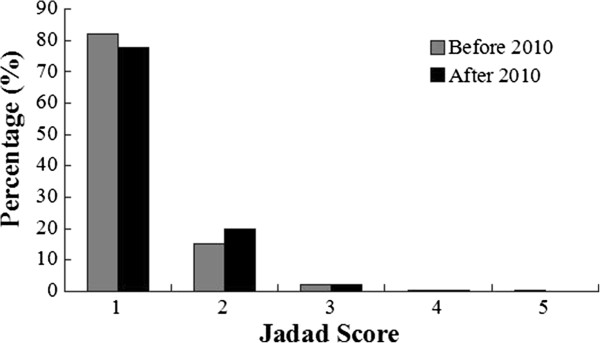
**The Jadad score before and after 2010.** The scores of reports are similar for both periods (2005–2009 vs 2011–2012, *P* = 0.405).

**Figure 11:**
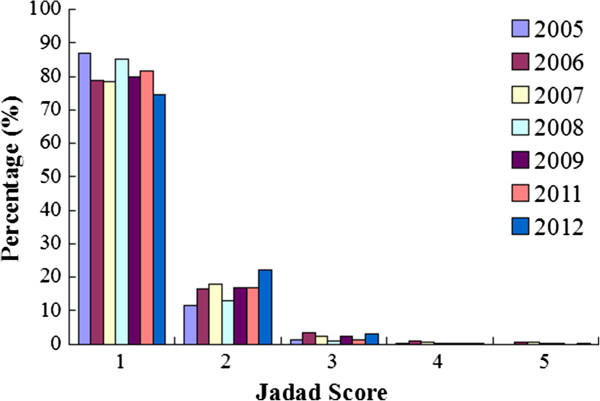
**The annual distributions of the Jadad score of reprts.** The mean scores are similar from 2005 to 2012 (*P* =1.000)

There are very few papers with a score above 2. The mean scores of reports are similar for both periods (1.22 for 2005–2009 vs 1.25 for 2011–2012, *P* = 0.405, Figure 10). The annual mean scores are similar from 2005 to 2012 (*P* = 1.000, Figure 11).

## Discussion

In the present study, we demonstrate that proportions of reports with descriptions of CONSORT items 1a, 2a, 4a, 4b, 8a, 12, 14a, 15 and 17b increase after 2010, while proportions of reports with descriptions of CONSORT items 1b, 2b, 3a, 10, 11a, and 25 decrease after 2010. And for most of the items, there is a fluctuation of proportion on description of the item from 2005 to 2012. These data indicate that publication of CONSORT has little, if any, influence on the most of the researchers reporting clinical trials in China.

TCM has been practiced in China for thousands of years. TCM doctors use herbal medicine to treat a variety of diseases. The medical herbs may be used singly or in combination. In the past decades, the effects of TCM have been evaluated in various animal models and the underlying mechanisms have also been explored in cellular, protein or DNA levels. Nevertheless, the efficiency of TCM should be demonstrated in RCTs, which is the top-level evidence for therapy. For example, Chansu, the skin and parotid venom glands of *Bufo bufo gargarizans cantor*, is a well-known TCM widely used for the treatment of a variety of tumors in China [34, 35]. Experimental studies suggested that Chansu and its active compounds exhibit significant anti-tumor activity via inhibiting cell proliferation, inducing apoptosis and cell arrest and inhibiting angiogenesis [36]. Further studies demonstrated that bufalin, one compound in Chansu, induced apoptosis of gastric cancer cells by inhibition of AKT signaling pathway [37] and inhibiting proliferation of hepatocellular carcinoma cells through inhibiting AKT/GSK3β/β-catenin/E-cadherin signaling pathway [38].

RCTs for TCM were first published in the 1980s [39]. Since then, a number of TCM RCTs have been published. However, the quality of the reports of the TCM RCTs were poor [39–44]. For example, Fang *et al.* reported that only 13 trials in 338 RCTs reports had the detailed description on method of randomization [40]. In the present study, we identified that only 8 of 31 CONSORT items have significant improvements from 2005–2009 to 2011–2012. A detailed and informative introduction of background can make readers understand the purpose of the study. Detailed inclusion and exclusion criteria of patients will avoid the selection bias. Clear and definite description of intervention is critical for the study to be repeated. In particular, whether outcome assessments are blind has considerable implications for assessment of internal validity [45]. We found that 29.00% of the articles described the background from 2005 to 2012. Sequence generation was described in only 16.26% of the publications, blinding in 3.83% and calculation of simple size in 0.29%. Inadequate description of these items will make the results of the study incredible. Another problem was there were only 56 out of 6994 reports that had the term ‘randomize’ in their titles. Title is a very important part of an article. Researchers use title to screen potential studies in meta-analysis.

With regard to methodological items, calculation of sample size was done by only 20 reports out of 6994 reports. If the sample is too large, it would be a waste of time and money. The smaller number of patients will reduce statistical power and generate selection bias. There were 268 reports using blinding method. The proportion of description on blinding method decreased after 2010. Blinding, especially double-blinding, is challenging for studies in which the intervention is being randomized [46]. Inadequate measures to create and conceal the random allocation, selective attrition, and insufficient double-blinding have been theorized to bias the estimates of treatment effects in RCTs [47].

Reports on adverse events were obvious not detailed enough, which will overestimate the safety of TCM. In fact, the recorded information of TCM herbs in most classical books includes toxicities, incompatibilities between herbs, cautions, precautions and contraindications. Thus, contrary to a general misconception, toxicity data on Chinese herbs exist and are documented through clinical experience [48]. For example, cinnabar, which contains mercury sulfide, has been used in TCM for thousands of years and 40 cinnabar-containing traditional medicines are still used today. Absorbed mercury from cinnabar is mainly accumulated in the kidneys, resembling the disposition pattern of inorganic mercury. Following long-term use of cinnabar, renal dysfunction may occur [49].

In addition, the reporting of outcomes and ancillary analyses remained poor. For example, intention-to-treat analysis is advocated because it preserves the randomization process and allows for noncompliance and deviations from policy by clinicians [50]. There are only 9 in 6994 papers using intention-to-treat analysis.

Discussion is an important part of a report. The author(s) can discuss the advantages and generalizability of the treatment, as well as the limitations of the study there. As we noticed, few reports have an informative discussion (Table 2). Finally, only one report contained information about registration, another one report contained information of protocol.

According to Jadad scale, there were 188 reports which scores were over 2 points. There was no difference between publications before and after 2010. Thus, reporting of TCM RCTs improved very slowly in their quality. The average Jadad score was 1.25 during 2011–2012, compared to 1.22 during 2005–2009.

In the present study we chose the CNKI database as the database to avoid selection bias. The CNKI database is the most comprehensive database in China. It achieves the full-text publications of 1217 medical Chinese journals, including 26 journals for TCM and 18 for integrative TCM and modern Western medicine. In addition, two researchers assessed independently the quality of each report by reading its full text. This is in sharp contrast to the previous reports, which evaluated only one or several TCM journals, or evaluated publication on a specific disease [24–29]. Thus the present study is the most comprehensive one on TCM RCTs.

Interestingly, we found that none of the manuscripts described change of the methods after trial commencement (Subitem 3b), change of trial outcomes after the trial commenced (Subitem 6b), interim analyses and stopping guidelines (Subitem 7b), premature discontinuation of the trial (Subitem 14b) and additional analyses (Subitem 18). The underlying reason is unknown.

## Conclusions

Although some improvements have been made in reporting TCM RCTs, the pace remains slow. And there remains considerable room for further improvement. The problems include optimal design of randomization, the usage of blinding, the calculation of sample size, comparability of baseline information, the clear and definite inclusion and exclusion criteria, the usage of statistical method, the withdrawal and follow-up of patients and the records of adverse events. Doctors practicing TCM should be trained to write high-quality reports and active implementation of the CONSORT guidelines by journals is necessary to make the reports on TCM RCTs more credible and TCM be used more widely in the world as an alternative medicine. We also suggest that a bibliographic database of TCM RCTs, similar to AcuTrials(R), be developed to enhance the accessibility and quality of TCM RCTs [51].

## References

[1] Katz JN, Wright JG, Losina E (2011). Clinical trials in orthopaedics research. Part II. Prioritization for randomized controlled clinical trials. J Bone Joint Surg Am.

[2] Schulz KF, Altman DG, Moher D (2011). CONSORT 2010 statement: updated guidelines for reporting parallel group randomised trials. Int J Surg.

[3] Chan AW, Altman DG (2005). Epidemiology and reporting of randomised trials published in PubMed journals. Lancet.

[4] Glasziou P, Meats E, Heneghan C, Shepperd S (2008). What is missing from de-scriptions of treatment in trials and reviews?. BMJ.

[5] Dwan K, Altman DG, Arnaiz JA, Bloom J, Chan AW, Cronin E, Decullier E, Easterbrook PJ, Von Elm E, Gamble C, Ghersi D, Ioannidis JP, Simes J, Williamson PR (2008). Systematic review of the empirical evidence of study publication bias and out-come reporting bias. PLoS One.

[6] Moher D, Hopewell S, Schulz KF, Montori V, Gotzsche PC, Devereaux PJ, Elbourne D, Egger M, Altman DG (2010). CONSORT 2010 explanation and elaboration: updated guidelines for reporting parallel group randomised trials. J Clin Epidemiol.

[7] Begg C, Cho M, Eastwood S, Horton R, Moher D, Olkin I, Pitkin R, Rennie D, Schulz KF, Simel D, Stroup DF (1996). Improving the quality of reporting of randomized controlled trials: the CONSORT statement. JAMA.

[8] Moher D, Schulz KF, Altman DG (2001). The CONSORT statement: revised recommendations for improving the quality of reports of parallel-group randomized trials. Ann Intern Med.

[9] Huwiler-Muntener K, Juni P, Junker C (2002). Quality of reporting of randomized trials as a measure of methodologic quality. JAMA.

[10] Mills EJ, Chow TW (2003). Randomized controlled trials in long-term care residents with dementia: a systematic review. J Am Med Dir Assoc.

[11] Hahn S, Puffer S, Torgerson DJ, Watson J (2005). Methodological bias in cluster randomised trials. BMC Med Res Methodol.

[12] Hopewell S, Dutton S, Yu LM, Chan AW, Altman DG (2010). The quality of reports of randomised trials in 2000 and 2006: comparative study of articles indexed in PubMed. BMJ.

[13] Jadad AR, Moore RA, Carroll D, Jenkinson C, Reynolds DJ, Gavahan DJ, McQuay HJ (1996). Assessing the quality of reports of randomized clinical trials: is blinding necessary?. Control Clin Trials.

[14] Olivo SA, Macedo LG, Gadotti IC, Fuentes J, Stanton T, Magee DJ (2008). Scales to assess the quality of randomized controlled trials: a systematic review. Phys Ther.

[15] Tindle HA, Davis RB, Phillips RS, Eisenberg DM (2005). Trends in use of complementary and alternative medicine by US adults: 1997–2002. Altern Ther Health Med.

[16] Chen FP, Chen TJ, Kung YY, Chen YC, Chou LF, Chen FJ, Hwang SJ (2007). Use frequency of traditional Chinese medicine in Taiwan. BMC Health Serv Res.

[17] Chung V, Wong E, Woo J, Lo SV, Griffiths S (2007). Use of Traditional Chinese Medicine in the Hong Kong special administrative region of China. J Altern Complement Med.

[18] Burke A, Upchurch DM, Dye C, Chyu L (2006). Acupuncture use in the United States: Findings from the National Health Interview Survey. J Altern Complement Med.

[19] Wu C, Liao L, Yan X, Li M, Wu S, Wang J, Lin J, Li S, Gao L, DU J, Yang R (2013). Effects of Yangxue Qingnao Granules on chronic cerebral circulation insufficiency: a randomized, double-blind, double-dummy, controlled multicentre trial. Psychogeriatrics.

[20] Chen Y, Fu DY, Chen Y, He YM, Fu XD, Xu YQ, Liu Y, Feng XT, Zhang T, Wang WJ (2013). Effects of Chinese herbal medicine Yiqi Huaju Formula on hypertensive patients with metabolic syndrome: a randomized, placebo-controlled trial. J Integr Med.

[21] Wang TZ, Chen Y, He YM, Fu XD, Wang Y, Xu YQ, Yang HJ, Xue HL, Liu Y, Feng XT, Zhang T, Wang WJ (2013). Effects of Chinese herbal medicine Yiqi Huaju Qingli Formula in metabolic syndrome patients with microalbuminuria: a randomized placebo-controlled trial. J Integr Med.

[22] Zhang Y, Chang J, Chi HH, Mao B, Tang WF, Wang L, Huang SZ, Li TQ, Zhang RM (2007). Randomized controlled trial on treatment of bronchial asthma of qi-deficiency cold syndrome type by pingchuan yiqi granule. Chin J Integr Med.

[23] Wenjuan S, Yuehui Z, Wei L, Jing C, Ying Z, Ng EHY, Xiaoke W (2013). Effects of tanshinone on hyperandrogenism and the quality of life in women with polycystic ovary syndrome: protocol of a double-blind, placebo-controlled, randomised trial. BMJ Open.

[24] Bian ZX, Li YP, Moher D, Dagenais S, Liu L, Wu TX, Miao JX, Kwan AK, Song L (2006). Improving the quality of randomized controlled trials in Chinese herbal medicine, part I: clinical trial design and method ology. J Chin Integr Med.

[25] Bian ZX, Mohe RD, Dagenais S, Li YP, Liu L, Wu TX, Miao JX (2006). Improving the quality of randomize d controlled trials in Chinese herbal medicine, part II: control group design. J Chin Integr Med.

[26] Bian ZX, Moher D, Dagenais S, Li YP, Wu TX, Liu L, Miao JX, Song L, Zhang HM (2006). Improving the quality of randomized controlled trials in Chinese herbal medicine, part IV: applying a revised CONSORT checklist to measure reporting quality. J Chin Integr Med.

[27] Yu H, Han Y, Xu J, Zang M, Wen ZH (2006). Systematic review of randomized controlled trials on treating chronic bronchitis with traditional Chinese medicine. Tradit Chin Res Clin Pharmacol.

[28] Sun J, Han K, Qian SC, You S (2009). Assessment of randomized controlled trials published in the Chinese medical Journal from 2007 to 2008. Chin Med J.

[29] Xu L, Li J, Zhang MM, Ai CL, Wang L (2008). Chinese authors do need CONSORT: Reporting quality assessment for five leading Chinese medical journals. Contemporary Clin Trials.

[30] *China Knowledge Resource Integrated Database*. [http://oversea.cnki.net/kns55/default.aspx]

[31] Schulz KF, Altman DG, Moher D, Berlin JA, Boutron I, Devereaux PJ, Dickersin K, Elbourne D, Ellenberg S, Gebski V, Goodman S, Gøtzsche PC, Groves T, Grunberg S, Haynes B, Hopewell S, James A, Juhn P, Middleton P, Minckler D, Montori VM, Mulrow C, Pocock S, Rennie D, Schriger DL, Simera I, Wager E, Clarke M, Guyatt G (2010). CONSORT 2010 Statement: updated guidelines for reporting parallel group randomised trials. Trails.

[32] Moher D, Hopewell S, Schulz KF, Gφtzsche PC MV, Devereaux PJ, Elbourne D, Egger M, Altman DG (2010). CONSORT 2010 Explanation and Elaboration: updated guidelines for reporting parallel group randomised trials (Chinese version). J Chin Integr Med.

[33] Augestad KM, Berntsen G, Lassen K, Bellika JG, Wootton R, Lindsetmo RO (2012). Standards for reporting randomized controlled trials inmedical informatics: a systematic review of CONSORT adherence in RCTs on clinical decision support. J Am Med Inform Assoc.

[34] Meng Z, Yang P, Shen Y, Bei W, Zhang Y, Ge Y, Newman RA, Cohen L, Liu L, Thornton B, Chang DZ, Liao Z, Kurzrock R (2009). Pilot study of huachansu in patients with hepatocellular carcinoma, nonsmall-cell lung cancer, or pancreatic cancer. Cancer.

[35] Qin TJ, Zhao XH, Yun J, Zhang LX, Ruan ZP, Pan BR (2008). Efficacy and safety of gemcitabine-oxaliplatin combined with huachansu in patients with advanced gallbladder carcinoma. World J Gastroenterol.

[36] Qi F, Li A, Inagaki Y, Kokudo N, Tamura S, Nakata M, Tang W (2011). Antitumor activity of extracts and compounds from the skin of the toad Bufo bufo gargarizans Cantor. Int Immunopharmacol.

[37] Li D, Qu X, Hou K, Zhang Y, Dong Q, Teng Y, Zhang J, Liu Y (2009). PI3K/Akt is involved in bufalin-induced apoptosis in gastric cancer cells. Anticancer Drugs.

[38] Qiu DZ, Zhang ZJ, Wu WZ, Yang KY (2013). Bufalin, a component in Chansu, inhibits proliferation and invasion of hepatocellular carcinoma cells. BMC Complement Altern Med.

[39] Mao B, Wang G, Fan T, Chen XD, Liu J, Wang L, Chang J, Ma JX, Guo J, Fu JJ, Li TQ (2007). Assessing the quality of reporting of randomized controlled trials in traditional Chinese medicine. Chin J Evid-Based Med.

[40] Fang RH, Liao XY, Su QL, Li SQ, Deng XX (2005). Evaluation of papers with randomized control trials published in clinical medicine in recent 16 years. West China Medical J.

[41] Li RQ, Liao XY, Fang RH, Li Y, Li SQ, Wang L (2005). Methodological evaluation of randomized controlled clinical therapeutic trials published in Chinese National Medicine Journal in the past 20 years. West China Medical J.

[42] Chang J, Li TQ, Wan MH, Zhang Y (2006). Quality assessment for randomized controlled trials published in four Acta of Traditional Chinese Medicine. Chin J Evid-Based Med.

[43] Dagenais S, Tricco AC, Bian ZX, Huang WH, Moher D (2006). Critical appraisal of clinical studies in Chinese herbal medicine. J Chin Integrative Med.

[44] Jia YL, Huang FY, Zhang SK, Liang SW (2012). Assessment of the quality of randomized controlled trials in Treating Coronary Heart Disease by Chinese Patent Medicine. CJITWM.

[45] Higgins JPT, Altman DG, Higgins JPT, Green S (2009). Assessing risk of bias in included studies. Cochrane handbook for systematic reviews of interventions. Version 5.0.2.

[46] McCormick F, Cvetanovich GL, Kim JM, Harris JD, Gupta AK, Abrams GD, Romeo AA, Provencher CM (2013). An assessment of the quality of rotator cuff randomized controlled trials: utilizing the Jadad score and CONSORT criteria. J Shoulder Elbow Surg.

[47] Schulz KF, Chalmers I, Hayes RJ, Altman DG (1995). Empirical evidence of bias. Dimensions of methodological quality associated with estimates of treatment effects in controlled trials. JAMA.

[48] Leung AY (2006). Traditional toxicity documentation of Chinese Materia Medica—an overview. Toxicol Pathol.

[49] Liu J, Shi JZ, Yu LM, Goyer RA, Waalkes MP (2008). Mercury in traditional medicines: is cinnabar toxicologically similar to common mercurials?. Exp Biol Med.

[50] Hollis S, Campbell F (1999). What is meant by intention to treat analysis? Survey of published randomised controlled trials. BMJ.

[51] Marx BL, Milley R, Cantor D, Ackerman D, Hammerschlag R (2013). AcuTrials(R): an online database of randomized controlled trials and systematic reviews of acupuncture. BMC Complement Altern Med.

[CR52] The pre-publication history for this paper can be accessed here:http://www.biomedcentral.com/1472-6882/14/362/prepub

